# Editorial: Oxytocin: Control of Bone and Fat Mass and Metabolism

**DOI:** 10.3389/fendo.2016.00027

**Published:** 2016-03-30

**Authors:** Ez-Zoubir Amri

**Affiliations:** ^1^CNRS UMR 7277, iBV, INSERM U1091, University of Nice Sophia Antipolis, Nice, France

**Keywords:** oxytocin, bone, fat, osteoporosis, obesity, diabetes mellitus, osteoblasts, adipocytes

**The Editorial on the Research Topic**

**Oxytocin: Control of Bone and Fat Mass and Metabolism**

Osteoporosis and overweight/obesity constitute major worldwide public health burdens. Human life expectancy has increased continuously in industrialized countries. Aging is associated with immunosenescence, a decrease in hormonal secretion, lean mass, and bone mass, and an increase in fat accumulation. It has been reported that both obesity and osteoporosis are affected by genetic and environmental factors; bone remodeling and adiposity are both regulated through the hypothalamus and sympathetic nervous system (1–3). Furthermore, mesenchymal stem cell represents a common precursor for adipocytes and osteoblasts; adipose tissue and skeleton are known endocrine organs. Oxytocin (OT) belongs to the pituitary hormone family and regulates the function of peripheral target organs, including the mammary glands and smooth muscle of the uterus (4). Due to this property, it has commonly been used in other medical obstetrics without significant side effects (5). It also modulates a wide range of behaviors, such as social recognition, love, and fear (6–9). Of note, it has been established that OT pathway was involved in the physiology of bone remodeling by the analysis of OT and its cognate receptor knock-out mice (10). Furthermore, OTR-deficient mice exhibit disorders in several aspects of social behavior, bone defects, and develop late-onset obesity (10–14). Thus, OT emerges as a promising molecule in the treatment of osteoporosis and obesity as well as associated metabolic disorders such as type 2 diabetes.

The levels of OT decreased with age. The crosstalk between bone and energy metabolism has been clearly evidenced in the last years through the investigations on the role of leptin, osteocalcin, and other molecules. Several reports, published recently, show clearly that OT could play an important role in the control of bone and fat mass and their metabolism (15–21). In conclusion, there are growing evidences showing that administration of OT holds promise as a potential therapy for both osteoporosis and weight gain/obesity, and may represent the first therapy targeting these two diseases linked to aging and their associated pathologies such as diabetes and cardiovascular disorders.

In this research topic, we were able to assemble articles from prominent scientists in the field, which focused on different aspects of OT. It contains four reviews and two original research articles. The topic started with a mini review regarding the central role of OT and food intake (Klockars et al.) followed by an update on the relationship between fat and bone (Colaianni et al.), then a review on OT treatment in relation to leptin signaling (Altirriba et al.), analysis of OT treatment against diabetes and osteoporosis (Elabd et al.), and finally two research articles describing the sex-dependent effects of OT (Beranger et al.) and the importance of OT receptor in the control of thermoregulation (Kasahara et al.).

I would like to thank the contributors and the reviewers for their help in putting interesting manuscripts in this special research topic.

## Author Contributions

The author confirms being the sole contributor of this work and approved it for publication.

## Conflict of Interest Statement

The author declares that the research was conducted in the absence of any commercial or financial relationships that could be construed as a potential conflict of interest.
